# Seroprevalence of human papillomavirus-16, -18, -31, and -45 in a population-based cohort of 10 000 women in Costa Rica

**DOI:** 10.1038/sj.bjc.6601272

**Published:** 2003-09-30

**Authors:** S S Wang, M Schiffman, T S Shields, R Herrero, A Hildesheim, M C Bratti, M E Sherman, A C Rodriguez, P E Castle, J Morales, M Alfaro, T Wright, S Chen, B Clayman, R D Burk, R P Viscidi

**Affiliations:** 1 National Cancer Institute, Bethesda, MD 20892-7234, USA; 2 Proyecto Epidemiologico Guanacaste, FUCODOCSA, Costa Rica 1253-1007, Mexico; 3 College of Physicians and Surgeons of Columbia University, New York, NY 10032, USA; 4 Information Management Services, Inc., Silver Spring, MD 20904, USA; 5 Stanley Division of Developmental Neurovirology, Department of Pediatrics, Johns Hopkins University School of Medicine, Baltimore, MD 21205, USA; 6 Albert Einstein College of Medicine, Bronx, NY 10461, USA

**Keywords:** HPV, seroprevalence, cervical cancer, predictors

## Abstract

Human papillomavirus (HPV) seroprevalence and determinants of seropositivity were assessed in a 10 049-woman population-based cohort in Guanacaste, Costa Rica. Serologic responses based on VLP-based ELISA were obtained from the plasma collected at study enrollment in 1993/1994 for HPV-16 (*n*=9949), HPV-18 (*n*=9928), HPV-31 (*n*=9932), and HPV-45 (*n*=3019). Seropositivity was defined as five standard deviations above the mean optical density obtained for studied virgins (*n*=573). HPV-16, -18, -31, and -45 seroprevalence was 15, 15, 16, and 11%, respectively. Of women DNA-positive for HPV-16, -18, -31, or -45, seropositivity was 45, 34, 51, and 28%, respectively. Peak HPV seroprevalence occurred a decade after DNA prevalence; lifetime number of sexual partners was the key determinant of seropositivity independent of DNA status and age. DNA- and sero-positive women showed the highest risk for concurrent CIN3/cancer, followed by DNA-positive, sero-negative women.

Currently, detection of cervical human papillomavirus (HPV) infection often relies on the identification of HPV DNA in exfoliated cervicovaginal cells. However, because most HPV infections (including oncogenic types) are transient and clear within 2 years, HPV DNA detection is limited to identifying current infections (Ho *et al*, 1995). The prevalence of HPV DNA therefore underestimates the cumulative incidence of infection in a population, particularly where the majority of women will not develop persistent infection or cervical neoplasia. Measurement of serum antibody to HPV capsids (or virus-like particles (VLPs)) has been demonstrated as a useful although imperfect marker of past and cumulative HPV exposure, thus complementing the assessment of HPV DNA (Kirnbauer *et al*, 1994; Wideroff *et al*, 1995, 1999; Carter *et al*, 1996; Sasagawa *et al*, 1998; Touze *et al*, 2001).

As part of a large population-based study of the natural history of HPV infection and cervical neoplasia in Guanacaste, Costa Rica, we report here the population-based seroprevalence in Guanacaste women of four oncogenic HPV types (HPV-16, -18, -31, and -45) found in the majority of cervical cancers worldwide (Bosch *et al*, 1995; Munoz *et al*, 2003). The data presented document the baseline burden of HPV exposure in Guanacaste, Costa Rica. As a major objective, we determined the association of risk for concurrent diagnosis of CIN3/cancer with HPV assessed jointly by serology and PCR-based DNA testing. As only about half of all HPV-DNA-positive women are seropositive using available VLP assays (Kirnbauer *et al*, 1994; Le Cann *et al*, 1995), we also investigated determinants of seropositivity in our population.

## METHODS

### Study population

This study was conducted in an on-going population-based cohort study of 10 049 women in Guanacaste, Costa Rica (Herrero *et al*, 1997; Hildesheim *et al*, 2001) who were enrolled in 1993–1994 with the approval of the National Cancer Institute (NCI) and local institutional review boards; all participants provided written informed consent. Briefly, the cohort was a representative sample of the adult population based on selection by cluster sampling, with oversampling for cancer. Participation among eligible women exceeded 90%, and participants completed a risk factor questionnaire that addressed demographic, behavioural, and sexual practices. Women were screened using three cytologic and one visual test at enrollment; colposcopy referral with biopsy was performed for any abnormal or equivocal screening results of suspicious lesions. For this analysis, a histologic diagnosis of CIN3 or cancer (*n*=107) was considered as the reference standard of serious high-grade disease.

### Serological measurements

Plasma samples collected at study enrollment were tested for anti-HPV L1 antibodies at the Johns Hopkins Medical Institutions by a VLP-based enzyme linked immunosorbent assay (ELISA) for HPV-16, -18, -31, and -45. Of the 10 077 women enrolled in the Costa Rican cohort, results were obtained for VLP serologic responses to HPV-16 (*n*=9949), HPV-18 (*n*=9928), HPV-31 (*n*=9932), and HPV-45 (*n*=3019). Based on results from our on-going DNA testing for the cohort, serologic testing for HPV-45 was discontinued following 3040 assays due to the comparatively low DNA prevalence (<1%).

HPV VLPs were prepared in *Trichoplusia ni* (High Five™) cells (Invitrogen, Carlsbad, CA, USA) from recombinant baculoviruses expressing the L1 and L2 genes of HPV-16 or -31 or the L1 gene alone of HPV-18 or -45. Virus-like particles were purified from cell pellets by density-gradient ultracentrifugation and column chromatography techniques as described previously (Studentsov *et al*, 2002; Viscidi *et al*, 2003). For the VLP-based ELISA, 96-well flat bottom PolySorp plates (Nunc, Naperville, IL, USA) were coated overnight at 4°C with 0.4–0.5 *μ*g total VLP protein per millilitre of phosphate-buffered saline (PBS), pH 7.2. The plates were blocked at room temperature for 3 h with 0.5% (wt vol^−1^) polyvinyl alcohol, MW 30 000–70 000 (Sigma, St Louis, MO, USA) in PBS (0.5% PVA). The blocking solution was replaced with PBS and the plates were stored at −20°C until use. Before use and following each incubation step, the plates were washed four times with wash solution (PBS-0.05% Tween 20) in an automatic plate washer (Skanwasher 300, Skatron, Lier, Norway). Using a MultiPROBE II robotic liquid handling system (Packard Instruments, Meriden, CT, USA), plasma specimens were diluted 1 : 10 in 0.5% PVA and 10 *μ*l of the diluted plasma was added to the antigen-coated plate containing 90 *μ*l of 0.5% PVA and incubated at 37°C for 1 h. After extensive washing, antigen-bound immunoglobulin was detected with horseradish peroxidase-conjugated goat anti-human IgG, gamma chain specific (Zymed, San Francisco, CA, USA), diluted 1 : 4000 in 0.5% PVA, 0.025% Tween 20, 0.8% (wt vol^−1^) polyvinylpyrrolidone, MA 360 000 (Sigma) in PBS. After 30 min at 37°C, colour development was initiated by the addition of 2,2′-azino-di-(3-ethylbenzthiazoline-6-sulphonate) hydrogen peroxide solution (Kirkegaard and Perry, Gaithersburg, MD, USA). The reaction was stopped after 20 min by addition of 1% dodecyl sulphate and absorbance was measured at 405 nm, with a reference wavelength of 490 nm, in an automated microtitre plate reader (Molecular Devices, Menlo Park, CA, USA).

Specimens were tested in duplicate on separate plates, with retesting of specimens with results exceeding a preset, acceptable coefficient of variation (CV) of 25% if one of the replicate optical density (OD) values was greater than the cut point for seropositivity (>0.05 OD units). Retesting by CV, however, did not apply to samples where both OD values were lower than the seropositivity cut point. Each batch comprised approximately 2000–3000 specimens, including both intrabatch and interbatch reliability repeat specimens. Each batch also included specimens from random samples of 200 of the 573 virgins in the study population, which were used to calculate an ELISA cutoff.

### HPV DNA testing

Cervical cytologic specimens were tested for HPV DNA using L1 MY09/MY11 consensus primer methods (Herrero *et al*, 2000; Castle *et al*, 2002a). While HPV VLP measurements were conducted on the entire cohort, HPV DNA measurements excluded study virgins and women for whom pelvic exams were not conducted (Hildesheim *et al*, 2001); HPV testing results were thus available for 9165 women. Results were obtained for both HPV DNA and VLP serologic responses to HPV-16 (*n*=9112), HPV-18 (*n*=9102), HPV-31 (*n*=9105), and HPV-45 (*n*=2774).

### Statistical methods

Serology results were dichotomized as antibody positive or negative. The cutoff was calculated independently for each test batch, by comparison with the distribution of the values obtained for the concurrently tested virgins in that batch (*n*=200). We used an iterative statistical approach that excluded outliers in the distribution of virgin test results until no remaining value was greater than two standard deviations above the mean optical density of the virgin specimens in that batch. Seropositivity for each HPV type was then defined as five standard deviations above the mean OD obtained for the concurrently tested virgins (minus virgin outliers). Alternative classifications of seropositivity using a three standard deviation cutpoint, or a receiver operating curve (ROC) approach contrasting the virgins to known type-specific DNA positives yielded the same conclusions.

Overall, multiple-type, and single-type seroprevalence estimates are reported. The seroreactivity of each HPV VLP type to the other measured HPV types was determined by calculating and comparing prevalence odds ratios (ORs). To assess possible antigenic crossreactivity between types, prevalence ORs were calculated for HPV seroreactivity and DNA status of the same or other HPV types (HPV-16, -18, -31, -45). To further assess the type specificity of the serologic assay, we calculated prevalence ORs for seroreactivity of HPV-16, -18, -31, and -45 to DNA positivity of HPV-6, -11, -26, -33, -35, -39, -40, -42, -51, -52, -53, -54, -55, -56, -57, -58, -59, -61, -62, -66, -68, and -71.

To identify determinants of seropositivity, univariate associations for HPV-16, -18, and -31 seropositivity were assessed for the following behavioural and reproductive variables: smoking (former, current, number of cigarettes), alcohol intake, early age at first intercourse (defined as <16 years), number of sexual partners (lifetime, and recently defined as within the last year), number of pregnancies, number of live births, and use of oral contraceptives (OC). Study virgins were excluded from these analyses; we also do not report associations with HPV-45, due to the discontinuation of this assay. Odds ratio estimates with 95% confidence intervals were obtained to assess the magnitude and statistical significance of the associations between HPV seropositivity and HPV DNA positivity, and to identify determinants of HPV seropositivity; statistical significance was also obtained by *χ*^2^. We included in our final multivariate model, variables statistically significantly associated with seropositivity in our univariate models: age (<25, 25–34, 35–44, 33–64, 65+ years), number of lifetime sexual partners (1, 2–3, 4+), age at first intercourse (<16, 16–19, 20+ years), number of recent sexual partners (0, 1, 2+), smoking (never, former, current), and OC use (never, former, current).

To further characterise HPV exposure in the population, we assessed exposure using both serological and DNA-based measurements. We categorised exposure into four groups: (i) women both HPV sero- and DNA-negative, (ii) women sero-positive but DNA-negative, (iii) women sero-negative but DNA-positive, and (iv) women both sero- and DNA-positive. For each HPV type, we used logistic regression to assess the cross-sectional association (OR with 95% confidence intervals (CI)) between HPV exposure and CIN3/cancer, adjusted for age. All analyses were conducted on SAS 8.2 for Windows.

## RESULTS

The median age of the 9949 women for whom IgG antibodies to HPV VLPs were measured was 38 years (range: 18–97 years). Overall, seroprevalence for HPV-16, -18, -31, and -45 was 15, 15, 16, and 11%, respectively (Table 1

**Table 1:** Seroprevalence of HPV-16, -18, -31, and -45 in the Costa Rican population-based cohort

**HPV type**	**Seroprevalence**	**Only this type**	**This plus other type^a^**
16 (*n*=9949)	1533 (15.4%)	579 (5.8%)	954 (9.6%)
18 (*n*=9928)	1537 (15.5%)	626 (6.3%)	911 (9.2%)
31 (*n*=9932)	1638 (16.5%)	643 (6.5%)	995 (10.0%)
45 (*n*=3019)	335 (11.1%)	91 (3.0%)	244 (8.1%)

a Other defined as HPV-16, -18, and -31. HPV-45 was not included due to termination of assay.

). Of the 1533 women seropositive for HPV-16, 579 (38%) were seropositive only for HPV-16 and 954 (62%) were seropositive for HPV-16 in addition to HPV-18 and -31. Similarly, for women seropositive for HPV-18, -31, and -45, the minority (41, 39, and 27%) were seropositive only for that type.

As more than 60% of seropositive women were seropositive for multiple HPV types, we assessed type-specificity to distinguish between true multiple infections and potential crossreactivity of the assays. We assessed the association between seropositivity to one HPV type and that to another type (Table 2

**Table 2:** Prevalence ORs and 95% CIs of HPV-16, -18, -31, and -45 seroreactivity *vs* seroreactivity to other type

	**HPV type by serology**		
**HPV type**	**16**	**18**	**31**
**by serology**	**OR (95% CI)**	**OR (95% CI)**	**OR (95% CI)**
18	7.0 (6.2–7.9)		
31	8.5 (7.5–9.6)	7.1 (6.3–8.0)	
45	6.6 (5.1–8.4)	14.9 (11.4–19.4)	10.3 (8.0–13.3)

). Seroreactivity to HPV-16, -18, -31, or -45 was strongly associated with seroreactivity to the other measured HPV types. Prevalence ORs appeared similarly elevated, although highest for HPV-18 and -45 (OR: 14.9; 95% CI: 11.4–19.4), HPV-31 and -45 (OR: 10.3; 95% CI: 8.0–13.3), and HPV-16 and -31 (OR: 8.5; 95% CI: 7.5–9.6). Analyses restricted to single infections defined by concurrent DNA resulted in diminished but still significant associations (data not shown).

To further clarify whether these associations reflect concomitant infections, we calculated the association between HPV seropositivity and viral DNA positivity for the same type and for the three other HPV types. As shown in Table 3

**Table 3:** Prevalence ORs and 95% CIs of HPV-16, -18, -31, and -45 seroreactivity *vs* DNA-type positivity of the same type

	**HPV type by serology**			
**HPV type**	**16**	**18**	**31**	**45**
**by DNA**	**OR (95% CI)**	**OR (95% CI)**	**OR (95% CI)**	**OR (95% CI)**
16	*4.5 (3.6–5.6)*	1.9 (1.5–2.5)	2.0 (1.6–2.6)	1.0 (0.5–2.0)
18	1.6 (1.0–2.4)	*2.7 (1.8–3.9)*	1.9 (1.3–2.8)	2.5 (1.0–6.4)
31	2.6 (1.8–3.8)	2.2 (1.5–3.2)	*5.1 (3.6–7.3)*	1.8 (0.8–4.0)
45	2.1 (1.3–3.6)	1.7 (1.0–3.0)	2.4 (1.5–4.0)	*3.0 (1.2–7.2)*

, for all four HPV types measured, the magnitude of the association was highest for each HPV serotype and DNA of the same type. The prevalence OR for HPV-16 seropositivity and HPV-16 DNA positivity was 4.5 (95% CI: 3.6–5.6). For HPV-18, -31, and -45 seropositivity and DNA positivity, the prevalence ORs were 2.7 (95% CI: 1.8–3.9), 5.1 (95% CI: 3.6–7.3), and 3.0 (95% CI: 1.2–7.2), respectively. The type-specificity of the serologic assay appeared weakest for HPV-18 and -45. When analyses were restricted to single infections of the specified HPV type by serology or by DNA, the magnitude of risk for serology and DNA of the same type remained similarly elevated, while the magnitude of association for serology and DNA of a different type declined to null, although confidence intervals were widened by reduced numbers. Moreover, when the analyses were restricted to HPV-exposed women, defined as either HPV DNA- or sero-positive for any of the four HPV types measured, thus reducing confounding due to (levels of relevant) sexual behaviour, the magnitude of association for HPV DNA and serology of the same type became much stronger. Again, CIs were widened due to reduced numbers. We also assessed the association between HPV-16, -18, -31, and -45 seropositivity with other HPV DNA types for which data were available (HPV-6, -11, -26, -33, -35, -39, -40, -51, -52, -53, -54, -55, -56, -58, -59, -61, -66, -68, -70, -71, -73); null associations largely were observed between HPV seropositivity with these other HPV types (data not shown), supporting type-specificity of the four serologic assays.

In our population, HPV seroprevalence was significantly higher than HPV DNA prevalence, which was 3.5% for HPV-16, 1.3% for HPV-18, 1.4% for HPV-31, and 0.8% for HPV-45. As described in the Methods section, HPV DNA was not measured in study virgins; therefore, with the assumption that virgins are HPV DNA-negative (Kjaer *et al*, 2001), the HPV DNA prevalence rates would be even lower. Of women HPV DNA-positive for each respective type, 45% were seropositive for HPV-16, 34% were seropositive for HPV-18, 51% were seropositive for HPV-31, and 28% were seropositive for HPV-45, thus reflecting the understanding that not all women infected with HPV will seroconvert, as defined within the detection limits of our assays and our stringent cutpoint. Conversely, of women seropositive for HPV-16, only 10% were HPV DNA-positive; for HPV-18, -31, and -45 seropositive women, DNA positivity for their same HPV type was 3, 4, and 2%, respectively, reflecting the transient nature of HPV infection.

**Figure 1:**
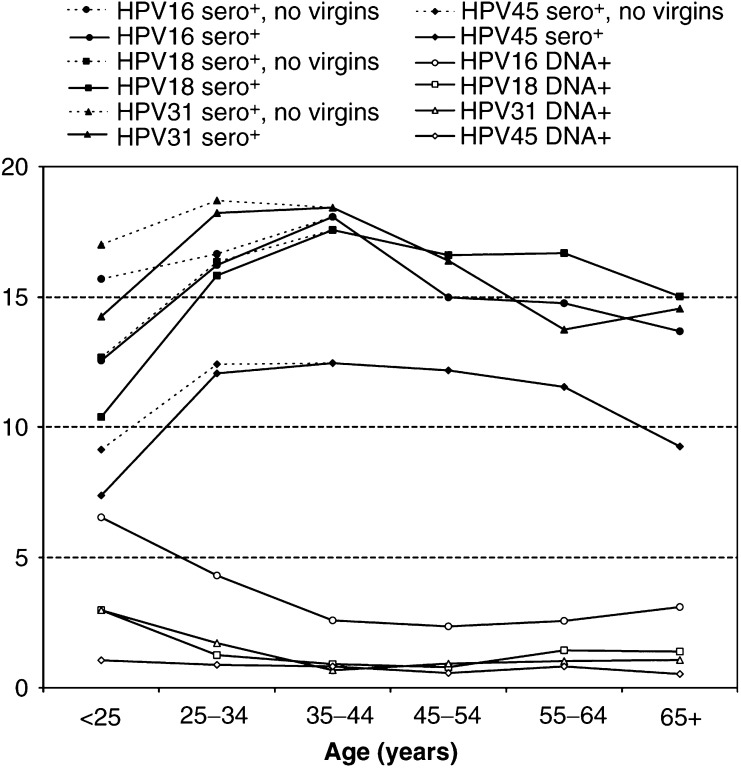
Age distribution of HPV-16, -18, -31, and -45 seroprevalence^*^ and DNA-prevalence^**^ in Guanacaste, Costa Rica women. ^*^Population-based HPV-16, -18, -31, and -45 serology prevalence is shown in black lines and includes study virgins. Black dotted lines denote seroprevalence excluding study virgins. ^**^HPV DNA prevalence does not include study virgins.

HPV seroprevalence and DNA prevalence by age are shown in Figure 1

. At all ages, HPV seroprevalence of all four types remained elevated compared to DNA positivity. HPV seroprevalence reached its highest peak at 25–34 years for HPV-31 and at 35–44 years for HPV-16, -18, and -45. Although seropositivity appeared to decline slightly with age after its peak, seroprevalence always remained elevated above the level seen in women less than 25 years old. In contrast, HPV DNA positivity peaked for the four HPV types in women less than 25 years old and declined with increasing age; for HPV-16, however, there appeared to be a slight secondary increase in DNA prevalence in the older age groups. It is important to note again that these data for HPV DNA positivity do not include virgins, the majority of whom were less than 25 years (64%) or 25–34 years (20%).

To identify determinants of HPV seropositivity, we assessed the association between demographic, social, and behavioural risk factors with HPV-16, -18, and -31 seropositivity in sexually active women (thus excluding virgins). Of all demographic and behaviuoral risk factors measured in univariate analyses, age, number of recent and past sexual partners, age at first intercourse, smoking, and OC use were statistically significantly associated with HPV seropositivity, and we therefore included them in our final multivariate model. As shown in Table 4

**Table 4:** Final logistic regression model demonstrating association between number of lifetime sexual partners, age at first sexual intercourse, smoking, OC use and HPV-16, -18, and -31 seropositivity, also adjusted for age and recent sexual partners (excluding study virgins)

	**HPV-16 seropositive**		**HPV-18 seropositive**		**HPV-31 seropositive**	
	**# (%)**	**OR (95% CI)**	**# (%)**	**OR (95% CI)**	**# (%)**	**OR (95% CI)**
*Age*						
<25	166 (15.7)	—	134 (12.7)	—	179 (16.9)	—
25–29	182 (14.2)	0.9 (0.7–1.1)	176 (13.7)	1.1 (0.8–1.4)	235 (18.3)	1.1 (0.9–1.4)
30–44	657 (18.4)	1.1 (0.9–1.4)	645 (18.1)	1.4 (1.2–1.7)	667 (18.7)	1.1 (0.9–1.3)
45–64	361 (15.0)	0.9 (0.7–1.2)	402 (16.7)	1.3 (1.0–1.7)	369 (15.3)	0.8 (0.7–1.0)
65+	143 (13.7)	0.9 (0.7–1.2)	157 (15.0)	1.3 (0.9–1.7)	152 (14.6)	0.8 (0.6–1.1)
						
*Lifetime number of sexual partners*						
1 (referent)	537 (10.6)	—	557 (11.0)	—	646 (12.8)	—
2–3	654 (20.3)	2.1 (1.8–2.3)	660 (20.5)	2.0 (1.7–2.2)	656 (20.4)	1.7 (1.5–1.9)
4+	318 (28.9)	3.1 (2.6–3.7)	297 (27.0)	2.6 (2.2–3.1)	300 (27.2)	2.3 (2.0–2.8)
						
*Number of recent sexual partners*						
0	297 (16.0)	—	315 (17.0)	—	305 (16.4)	—
1	1153 (15.8)	0.9 (0.8–1.1)	1134 (15.6)	0.9 (0.8–1.1)	1235 (16.9)	0.9 (0.8–1.1)
2+	58 (26.9)	0.9 (0.6–1.3)	65 (30.1)	1.3 (0.9–1.8)	60 (27.8)	1.0 (0.7–1.5)
						
*Age at first intercourse*						
<16 (referent)	415 (21.1)	—	419 (21.4)	—	403 (20.5)	—
16–19	726 (15.7)	0.8 (0.7–1.0)	718 (15.5)	0.8 (0.7–0.9)	816 (17.6)	1.0 (0.8–1.1)
20+	367 (13.3)	0.8 (0.7–1.0)	376 (13.6)	0.8 (0.6–0.9)	382 (13.8)	0.8 (0.7–1.0)
						
*Smoking*						
Never (referent)	1285 (15.5)	—	1304 (15.7)	—	1380 (16.6)	—
Former	111 (19.6)	1.2 (0.9–1.4)	105 (18.5)	1.0 (0.8–1.3)	114 (20.1)	1.2 (0.9–1.5)
Current	113 (22.9)	1.2 (1.0–1.5)	103 (20.9)	1.1 (0.8–1.3)	107 (21.7)	1.1 (0.9–1.4)
						
*Oral contraceptive use*						
Never (referent)	511 (13.9)	—	553 (15.1)	—	570 (15.5)	—
Former	671 (17.0)	1.3 (1.1–1.5)	683 (17.4)	1.2 (1.0–1.4)	708 (18.0)	1.1 (1.0–1.3)
Current	326 (18.5)	1.5 (1.2–1.8)	277 (15.7)	1.2 (1.0–1.4)	324 (18.4)	1.1 (0.9–1.3)

, there was a clear stepwise increase in prevalence and association with seropositivity for all four HPV types with increasing number of lifetime sexual partners. In our final model, the number of recent sexual partners, defined as within the past year, was no longer associated with HPV-16 seropositivity. Since having two or more recent partners was highly correlated with a high lifetime number of sexual partners, there was likely confounding by past exposure. Finally, for women who initiated sexual intercourse at older ages, statistically significant but small decreases in risk were observed for HPV-16, -18, and -31 seropositivity; this is likely due to the decreased lifetime number of sexual partners that is correlated with those initiating sexual intercourse at older ages.

As shown in Table 4, current smoking exhibited a borderline association with HPV-16 seropositivity, with a risk of 1.2 (95% CI: 1.0–1.5); former and current OC use were associated with HPV-16 seropositivity with risks of 1.3 (95% CI: 1.1–1.5) and 1.5 (95% CI: 1.2–1.8), respectively. No association was demonstrated between HPV-18 seropositivity and smoking, but borderline associations were observed for current OC use, and between HPV-31 seropositivity and former OC use. Taken together, these results support the role of sexual behaviour as the key determinant of HPV seropositivity; the HPV cofactors for cervical cancer, namely OC use, may play a minor role in the risk of HPV-16 seropositivity. Determinants for combined HPV-16, -18, or -31 seropositivity similarly included number of lifetime sexual partners and age at first intercourse. However, neither smoking nor OC use was associated with combined seropositivity (data not shown).

We assessed the risk of CIN3/cancer associated with HPV exposure as determined by HPV DNA and serology measurements for HPV-16, -18, and -31. As shown in Table 5

**Table 5:** Association between differential exposures (by serology and DNA) and disease outcome of CIN3/cancer, adjusted by age

**HPV**	**Serology**	**DNA**	**Total *n*^a^**	**CIN3/cancer *n* (%)**	**CIN3/cancer positive for another oncogenic type**	**OR (95% CI)**
16	+	+	144	21 (14.6)	4	34.7 (19.7–61.0)
	−	+	179	30 (16.8)	6	39.9 (24.1–66.2)
	+	−	1333	15 (1.1)	11	2.0 (1.1–3.7)
	−	−	7456	41 (0.5)	32	1.0
						
18	+	+	41	5 (12.2)	3	16.6 (6.3–43.9)
	−	+	80	8 (10.0)	3	13.6 (6.3–29.6)
	+	−	1436	25 (1.7)	21	1.9 (1.2–3.0)
	−	−	7545	68 (0.9)	59	1.0
						
31	+	+	63	7 (11.1)	3	14.1 (6.2–32.2)
	−	+	61	3 (4.9)	2	5.7 (1.7–18.7)
	+	−	1502	25 (1.7)	21	1.7 (1.1–2.7)
	−	−	7479	72 (1.0)	63	1.0

a Total *n*=9112; HPV-18 and HPV-31 exposure groups do not add up to 9112 due to missing data.

, HPV-16 DNA-positive women possessed the greatest magnitude of risk for CIN3/cancer, regardless of serologic status, with age-adjusted ORs of 34.7 (19.7–61.0) for women both HPV-16 DNA- and sero-positive, and 39.9 (05% CI: 24.1–66.2) for women only HPV-16 DNA-positive. Women only seropositive for HPV-16 possessed a much lower, but still significantly elevated level of risk for CIN3/cancer, with OR of 2.0 (age-adjusted OR: 1.1–3.7). For HPV-18 and -31, the magnitude of association for women both DNA- and sero-positive with CIN3/cancer was the highest, followed by women DNA-positive only. Women only seropositive for HPV-18 possessed a moderate increase in risk for CIN3/cancer with an age-adjusted OR of 1.9 (95% CI: 1.2–3.0); women only seropositive for HPV-31 also possessed an increase in risk for CIN3/cancer (age-adjusted OR: 1.7; 95% CI: 1.1–2.7). Of CIN3/cancers seropositive for either HPV-16, -18, or -31, and DNA-negative for the respective type, the majority were also DNA-positive for another measured oncogenic HPV type at the time of the serological measurement, although it is unknown whether these are the same HPV types found in the tumour. When the analyses are restricted to single infections as defined by seropositivity, the magnitudes of association were slightly diminished for women seropositive but DNA-negative for HPV-31, but stayed constant for HPV-16 and -18 seropositivity.

We also conducted analyses stratified by age to delineate clearly risk relationships among young women, because past and recent exposures are likely equivalent in this age group. For women less than 25 years of age, the highest level of risk for CIN3/cancer was observed for HPV-16 DNA-positive women (OR=41.9) rather than DNA- and sero-positive women (OR=26.2). Higher risk estimates in DNA-positive women less than 25 years of age were also demonstrated for HPV-18. No statistically significant associations were observed between women HPV seropositivity only and CIN3/cancer for women less than 25 years of age. Exclusion of women less than 25 years of age did not significantly change the results.

## DISCUSSION

Overall, the HPV seroprevalence of 15–16% in our population is consistent with the broad range (10–52%) found in previous reports (Nonnenmacher *et al*, 1996; Hagensee *et al*, 1999; Stone *et al*, 2002). HPV-16 alone accounts for approximately half of all cervical cancers in Guanacaste (Herrero *et al*, 2000) and worldwide (Munoz *et al*, 2003); likewise, the population-based prevalence of HPV-16 DNA positivity is highest in our cohort. However, this predominance of HPV-16 is not reflected in seroprevalence, and the overall and age-specific seroprevalence of HPV-16, -18, and -31 are equivalent, despite the known differences between these HPV types. The reason for the difference between type-specific DNA prevalence and seroprevalence is unclear. We used a stringent cutoff for defining seropositivity, which probably underestimated the seroprevalence in our population. However, the potential for assay crossreactivity may have resulted in an opposite effect of overestimating seroprevalence. Based on our analyses, we believe that these magnitudes of risk reflect multiple infections in our population, but crossreactivity cannot be ruled out. Although magnitudes of risk appeared highest for related HPV types (e.g., HPV-16 and -31, HPV-18 and -45), they were also not exclusive to related types (e.g., HPV-31 and -45). One possible explanation for the discrepancy between DNA- and sero-positivity may be the differential incidence and duration of type-specific HPV.

The differential peaks for HPV DNA- and sero-prevalence are of interest. It is well understood that high DNA positivity rates in young women are due to sexual activity. While HPV seroconversion can take up to 18 months (Carter *et al*, 1996, 2000), in Guanacaste, seroprevalence peaked 10–20 years later. This decade-long lag time is probably due to the low seroconversion rate and/or the potential need for repeated exposures and thus, antigenic stimulation, to induce a detectable antibody response. The simultaneous increase in seroprevalence and decline in DNA prevalence in women over 25 years of age may indicate that these women continue to be exposed to HPV as a result of repeated or recurrent infection or reactivation of latent infection, but that levels of viral replication are below the detection limit of PCR. However, the slight secondary increase in HPV-16 DNA prevalence in the older age groups is noted and previously reported (Herrero *et al*, 2000). The seroprevalence peak is also 10–20 years after sexual initiation, thus potentially mirroring the effect of multiple lifetime sexual partners, which correlates with age. However, unlike other studies, we did not observe an increase in HPV seropositivity with age (Strickler *et al*, 1999; Studentsov *et al*, 2002). On the contrary, the slight decrease of seropositivity observed in our older age groups supports the suggested waning of seropositivity over time and the imperfect nature of serology as a measure of cumulative exposure; alternatively, this could reflect a cohort effect.

The moderate overall agreement between HPV seropositivity and DNA positivity in our study is consistent with other studies (Olsen *et al*, 1996), further confirming that only a subset of women exposed to HPV will seroconvert. Although we comprehensively assessed demographic, social, and behavioural variables to identify determinants of seropositivity, our findings support the single overriding factor to be exposure to HPV as measured by past sexual activity, as others have observed (Dillner *et al*, 1996; Andersson-Ellstrom *et al*, 1996; Wideroff *et al*, 1996; Kjellberg *et al*, 1999; Kjaer *et al*, 2001; Castle *et al*, 2002b;). Our data shown in Table 4 demonstrate that for HPV-16, -18, and -31, there are stepwise increases in risk of CIN3/cancer with increasing lifetime number of sexual partners, and decreases in risk of CIN3/cancer with increasing age of first sexual intercourse. These associations were also clearly observed in our subset of women with HPV-45 serology measurements. Only for HPV-16 seropositivity were the additional determinants, OC use, found to be statistically significant; these were borderline to not statistically significant for HPV-18 and -31 seropositivity. For HPV-16, our results support those by Stone *et al* (2002), who also demonstrate elevated risk of HPV-16 seropositivity with OC use (ever). Although Shin *et al* (2003) demonstrated a modest association between smoking and HPV seropositivity, the associations for HPV-16, -18, and -31 in our data were borderline or null. The effects of these cofactors, even if real, are likely modest at best.

A number of studies have supported the use of HPV-16 VLP as a biomarker of past HPV exposure, with moderate associations between HPV seropositivity and cervical cancer (Nonnenmacher *et al*, 1995; Dillner *et al*, 1996). As previously reported, the magnitude of association between seropositivity and cervical cancer in this study were 2–3-fold compared to HPV seronegative women (Nonnenmacher *et al*, 1995; Chua *et al*, 1996; Dillner *et al*, 1996, 1997; Wideroff *et al*, 1996; Wang *et al*, 2000; Sigstad *et al*, 2002). Our stratified analyses clearly demonstrated that HPV DNA-positive women had the highest levels of risk for CIN3/cancer; for HPV-18 and -31, risk for CIN3/cancer was highest for women both HPV DNA- and sero-positive followed by women DNA-positive only. While we observed a moderate risk of two-fold for HPV-16, -18, and -31 seropositivity in the absence of HPV DNA positivity in cross-sectional analyses, the majority of these women were seropositive (HPV-16, -18, -31, or -45) or DNA-positive for another measured oncogenic HPV type. However, to assess reliably whether the independent association between seropositivity and disease was confounded by HPV infection would require HPV typing in the histologic tissue. Finally, although previous studies have reported an increasing association of HPV seropositivity with disease severity (De Sanjose *et al*, 1996; Heim *et al*, 2002), this was not observed in our study. Stratification of disease by CIN1, CIN2, CIN3, and cancer did not demonstrate a stepwise increase in association, although risk for CIN3 and cancer did exceed that for CIN1 and CIN2 (data not shown).

To our knowledge, this is the largest population-based seroprevalence study of the four major HPV types: HPV-16, -18, -31, and -45; subjects were representative of the adult female population of Guanacaste, Costa Rica. Although serology measurements underestimate cumulative exposure to HPV due to low seroconversion and possibly waning seropositivity over time, detection of serum VLP antibodies to HPV nevertheless provides an informative assessment of HPV exposure in a population. To understand the implications of HPV serology in the natural history of cervical cancer, however, will require prospective studies.
